# VLDL Hydrolysis by Hepatic Lipase Regulates PPARδ Transcriptional Responses

**DOI:** 10.1371/journal.pone.0021209

**Published:** 2011-07-05

**Authors:** Jonathan D. Brown, Eric Oligino, Daniel J. Rader, Alan Saghatelian, Jorge Plutzky

**Affiliations:** 1 Division of Cardiovascular Medicine, Brigham and Women's Hospital, Harvard Medical School, Boston, Massachusetts, United States of America; 2 VA Boston Healthcare, West Roxbury, Massachusetts, United States of America; 3 Division of Cardiology, Yale-New Haven Hospital, New Haven, Connecticut, United States of America; 4 Department of Medicine, Hospital of the University of Pennsylvania, Philadelphia, Pennsylvania, United States of America; 5 Department of Chemistry, Harvard University, Cambridge, Massachusetts, United States of America; Chinese University of Hong Kong, Hong Kong

## Abstract

**Background:**

PPARs (α,γ,δ) are a family of ligand-activated transcription factors that regulate energy balance, including lipid metabolism. Despite these critical functions, the integration between specific pathways of lipid metabolism and distinct PPAR responses remains obscure. Previous work has revealed that lipolytic pathways can activate PPARs. Whether hepatic lipase (HL), an enzyme that regulates VLDL and HDL catabolism, participates in PPAR responses is unknown.

**Methods/Principal Findings:**

Using PPAR ligand binding domain transactivation assays, we found that HL interacted with triglyceride-rich VLDL (>HDL≫LDL, IDL) to activate PPARδ preferentially over PPARα or PPARγ, an effect dependent on HL catalytic activity. In cell free ligand displacement assays, VLDL hydrolysis by HL activated PPARδ in a VLDL-concentration dependent manner. Extended further, VLDL stimulation of HL-expressing HUVECs and FAO hepatoma cells increased mRNA expression of canonical PPARδ target genes, including adipocyte differentiation related protein (ADRP), angiopoietin like protein 4 and pyruvate dehydrogenase kinase-4. HL/VLDL regulated ADRP through a PPRE in the promoter region of this gene. *In vivo*, adenoviral-mediated hepatic HL expression in C57BL/6 mice increased hepatic ADRP mRNA levels by 30%. In ob/ob mice, a model with higher triglycerides than C57BL/6 mice, HL overexpression increased ADRP expression by 70%, demonstrating the importance of triglyceride substrate for HL-mediated PPARδ activation. Global metabolite profiling identified HL/VLDL released fatty acids including oleic acid and palmitoleic acid that were capable of recapitulating PPARδ activation and ADRP gene regulation *in vitro*.

**Conclusions:**

These data define a novel pathway involving HL hydrolysis of VLDL that activates PPARδ through generation of specific monounsaturated fatty acids. These data also demonstrate how integrating cell biology with metabolomic approaches provides insight into specific lipid mediators and pathways of lipid metabolism that regulate transcription.

## Introduction

Peroxisome Proliferator-Activated Receptors (PPARs) are ligand-activated transcription factors that regulate genes involved in energy balance [1]. Three PPAR isotypes – alpha (α), beta/delta (β/δ), and gamma (γ)- are expressed in metabolically active tissues as well as vascular and inflammatory cells [2], [3]. Genetic models and studies with synthetic PPAR agonists reveal key but distinct functions for PPARα and PPARδ in regulating fatty acid (FA) metabolism in muscle and liver, whereas PPARγ is a critical determinant of adipose tissue differentiation [4]. PPAR activation also modulates inflammatory pathways relevant for atherosclerosis and type 2 diabetes (T2D) [5]–[8]. Although various lipid mediators including long chain FAs, eicosanoids and phospholipids (PLs) are reported to activate PPARs *in vitro*, insight into pathways that can generate endogenous PPAR ligands has been limited [9], [10]. In the absence of such information, it remains difficult to place PPAR activation and subsequent transcriptional responses into a physiologic or pathologic network.

Prior work suggests that lipase-mediated lipoprotein metabolism can regulate PPAR responses. Both lipoprotein lipase (LPL) and endothelial lipase (EL) preferentially activate PPARα through hydrolysis of VLDL and HDL, respectively [11]–[14]. The specific LPL and EL-generated metabolites that mediate these responses have remained unidentified. More recently, hepatic *de novo* lipogenesis was reported to produce an endogenous phospholipid PPARα ligand in murine liver with no effect on PPARδ or PPARγ [10]. Since all three PPAR isotypes are expressed in hepatocytes, the selectivity of *de novo* lipogenesis for PPARα activation suggests that other pathways of lipid metabolism in the liver may be involved in PPARδ or PPARγ activation.

Hepatic lipase (HL), expressed in hepatocytes as well as macrophages, is central to lipoprotein metabolism [15]–[17]. As both a triacylglycerol hydrolase and phospholipase, HL has been shown to metabolize HDL, IDL and VLDL substrates, yielding FAs as well as other lipid mediators [18]. Murine transgenic and HL-deficiency models have established that HL regulates HDL and IDL-cholesterol with modest effects on VLDL triglyceride (TG) content [19], [20]. Humans carrying an HL loss-of-function mutation manifest elevated TG content in lipoproteins including VLDL and HDL [21]. Despite these important effects, uncertainty persists regarding HL's role in systemic metabolism. Indeed, HL has been alternatively reported to promote or limit both atherosclerosis and T2D [22]–[26]. Transcriptional responses induced through HL action have not been previously explored.

We postulated that HL hydrolytic activity might be involved in transcriptional regulation via PPARs, given the role of these FA-activated nuclear receptors in hepatic responses. We also reasoned that probing HL's effects on transcriptional regulation might provide a new way to consider functional roles of HL in systemic metabolism. In contrast to EL and LPL, which activate PPARα, we demonstrate here that HL hydrolyzes VLDL to generate predominantly PPARδ activation. By integrating this data with a global metabolite profiling approach, we found that VLDL hydrolysis by HL generates specific unsaturated FAs that can induce canonical PPARδ dependent transcriptional responses *in vitro* and *in vivo*, with functional effects on cellular lipid droplet formation in hepatocytes. Together these data provide new insight into HL biology, how PPARδ may be activated and an example of how coupling cell biology with metabolomic methods can identify specific lipid mediators exerting distinct biologic effects.

## Materials and Methods

### Ethics statement

All animal experiments were approved by the Institutional Animal Care and Use Committee (IACUC) and conducted in agreement with NIH policy (Protocol # 03121).

### Reagents

A 1.5 kb human HL cDNA was subcloned into pcDNA3 [18]. The HL catalytic mutant was generated by replacing serine position 149 with alanine using the Stratagene QuikChange PCR approach (primers 5′-CACCTAATTGGGTACGCCCTGGGTGCA-3′ and 5′CGTGTGCACCCAGGGCGTACCCAATTA-3′), and the mutation was confirmed by DNA sequencing. Adenovirus HL catalytic mutant was amplified/purified by Welgen, Inc. (Worcester, MA). Human lipoproteins (VLDL, HDL) were purchased from Biomedical Technologies, Inc (Stoughton, MA). LDL was isolated by potassium bromide density ultracentrifugation [11]. IDL was prepared from plasma of healthy volunteers as previously described. Lipoprotein concentrations are normalized to protein in µg/mL and stimulations were performed for each lipoprotein fraction at levels consistent with the published literature. [23], [27]–[29]. Chemicals were purchased from: Roche Pharmaceutics (Tetrahydrolipstatin), Alexis Biochemical (GW501516), Cayman Chemical (WY14643 and all FAs), Sigma-Aldrich (Lipoprotein deficient serum, triolein, egg phosphatidylcholine and FA-free BSA). The time-resolved, fluorescence resonance energy transfer (TR-FRET) PPARδ competitive binding assay was performed for PPARδ as per manufacturer protocol (Invitrogen) using a PerkinElmer Envision fluorescence plate reader. Briefly, a GST tagged recombinant PPARδ-LBD is incubated with a terbium labeled anti-GST antibody along with a fluorescein labeled small molecule (synthetic) PPAR ligand. In the absence of exogenous ligand, the fluorescein labeled PPAR ligand binds to the PPAR-LBD and FRET occurs between the terbium and fluorescein fluorophores. In the presence of an unlabeled PPAR ligand, displacement of the fluorescein PPAR ligand reduces FRET as measured by the emission ratio of 520 nm/495 nm.

### Cell culture

FAO hepatoma cells were maintained in RPMI supplemented with 10% FBS and antibiotics. HUVEC were cultured in M-199 medium supplemented with 20% fetal bovine serum, endothelial cell growth factor, 1% heparin and penicillin/streptomycin antibiotic. Lipoprotein stimulations were done in serum free medium. Fatty acid-BSA complex was generated by mixing oleic acid with 10%, fatty acid free BSA (4∶1 molar ratio) for 1 hour at 37 degrees.

### Cell transfection, reporter assays, siRNA and adenovirus infection

HEK-293, COS-7 and FAO cells were transfected in a 24 well plate with each PPAR-ligand binding domain construct (LBD) using Fugene HD (Roche) as previously described. [12], [30]. Briefly, fusion constructs of the PPAR-LBDs and the yeast GAL4 DNA binding domain were transfected into cells at 100 ng DNA/well along with a GAL4 dependent luciferase reporter at 70 ng DNA/well and the internal control CMV-β galactosidase at 50 ng DNA/well. Where indicated, cells were also transfected with human HL, catalytically inactive HL or control vector (pcDNA3) at 100 ng DNA/well. PPAR ligand activity was quantified by changes in luciferase reporter gene expression measured by standard luminometer assay and normalized to β-galactosidase. ADRP promoter constructs were a gift from Dr. Toshiya Tanaka, University of Tokyo, Japan [31] and were transfected at 100 ng DNA/well in a 24 well plate. SiRNA constructs (Ambion) were transfected using siDeliverX reagent (Panomics) according to manufacturer's protocol. For adenovirus experiments, cells were seeded (1×10^6^) in 6 well plates and infected for 24 hours with adenovirus constructs (MOI 100) in 1 mL of serum free medium. The media was replaced with fresh, serum free media with indicated concentrations of lipoproteins for 12 hours unless otherwise indicated.

### Mice

Eight-week-old male, C57BL/6 and ob/ob mice were purchased from Jackson Laboratories (Bar Harbor, ME). Animals were acclimated for 5 days and fed standard chow diet. Adenovirus was injected by tail vein using 1×10^11^ virus particle in 100 µL volume. Five days after injection, mice were euthanized and whole liver, epididymal fat and quadriceps skeletal muscle were isolated and snap frozen in liquid nitrogen for RNA isolation.

### RNA isolation, reverse transcription and real time PCR

Total cell mRNA was isolated using an RNeasy kit (Qiagen). For whole liver and skeletal muscle RNA isolation, 30–50 micrograms of tissue were homogenized in RNA lysis buffer 1 mL (Qiagen); epididymal fat from wild type mice and ob/ob liver RNA isolation was isolated using the RNeasy lipid isolation kit (Qiagen). Following isolation, 1 microgram of RNA was digested with DNase I (invitrogen) and reverse transcribed using QuantiTect kit (Qiagen). Real Time PCR (RT-PCR) was performed in a MyiQ Single-Color Real Time PCR system using SYBR Green I (Bio-Rad). The mRNA levels of test genes were normalized to 36B4 internal control gene. Standard curves were generated to calculate relative copy number. The primer sequences used were: Human ADRP: 5′-TGAGATGGCAGAGAACGGTGTG-3′ and 5′-GGCATTGGCAACAATCTGAGT-C-3′; Human 36B4: 5′-CAACCCAGCTCTGGAGAAAC-3′ and 5′-GTGAGGTCCTCCTTGGTGAA-3′; Human PPARδ: 5′-TGGCTTTGTCACCCGTGAGT-3′ and 3′-ACTGAGTTCGCCAAGAGCAT-3′;Rat ADRP: 5′-GCCTCTCAACTGGCTGGTAG and 5′-GCTCAGACTGCTGGACCTTC-3′; Rat 36B4: 5′-CACCTTCCCACTGGCTGAA-3′ and 5′-TCCTCCGACTCTTCCTTTGC-3′; Murine ADRP: 5′-CAAGTCGGAGCTGCTGGTAG-3′ and 5′-CCGAGAGCAGAGCTTGGTAG-3′: Murine 36B4: 5′-CAACCCAGCTCTGGAGAAAC-3′ and 5′-GAGGTCCTCCTTGGTGAACA-3′.

### Western blotting

Transfected cells were lysed in RIPA buffer containing EDTA and protease inhibitors. Following SDS-PAGE separation, immunoblotting was performed using a monoclonal antibody human HL antibody (Santa Cruz Biotechnology) as described previously [32]. Human HL western was performed on plasma following a 50-fold dilution in PBS.

### HL activity assay

A glycerol-stabilized emulsion of triolein and egg phosphatidylcholine containing glycerol-tri[9,10(n)-^3^H] oleate was used to measure HL activity in conditioned media from cells as described previously [32]. For HL activity *in vivo*, plasma was collected at baseline or 30 minutes following heparin injection (intraperitoneal, 200 U); activity assays were performed on 10 microliters of plasma.

### Liquid Chromatography/Mass Spectrometry

For metabolite profiling experiments, COS-7 cells (1×10^6^) were infected with adenoviral GFP or HL- expressing constructs for 24 hours in 6 well plates. Cells were treated with heparin 10 U/mL for 1 hour, followed by stimulation with VLDL at 50 µg/mL for 6 hours. The cell supernatant was harvested on ice, centrifuged at 3000 rpm for 5 minutes, transferred to a fresh tube and snap frozen in liquid nitrogen for subsequent lipid extraction. A 2∶1∶1 CHCL_3_/MeOH/conditioned media solution was prepared for lipid extraction to isolate organic soluble metabolites. A ^13^C oleic acid standard was included in these samples for targeted FA quantification. Following brief vortexing, samples were centrifuged at 2500 rpm at 4 degrees for 10 minutes. After centrifugation the organic layer (bottom) was transferred to a new vial and solvents evaporated under a stream of nitrogen. Samples were resuspended in CHCL_3_ (120 µL) and stored at −80 until LC/MS analysis (within 48 hours of extraction). For both positive and negative ionization mode LC/MS runs, 30 µl of extract was injected.

LC/MS analysis was performed using an Agilent 6530 Accurate-Mass Quadrupole-TOF LC/MS system. For LC analysis in negative mode, a Gemini (Phenomenex) C18 column (5 mm, 4.6×100 mm) was used together with a pre-column (C18, 3.5 mm, 2×20 mm). Mobile phase A consisted of 95/5 water/methanol and mobile phase B was composed of 60/35/5 isopropanol/methanol/water. Both A and B were supplemented with 0.1% ammonium hydroxide. The flow rate for each run was 0.5 µL/min. The gradient started at 0% B and linearly increased to 100% B over 40 minutes, was then maintained at 100% B for 8 minutes before equilibrating for 8 minutes at 0% B. For the LC analysis in positive mode, a Luna (Phenomenex) C5 column (5 mm, 4.6×100 mm) was used together with a pre-column (C4, 3.5 mm, 2×20 mm). Mobile phase A and B and the gradient were the same as for negative mode, but supplemented with 0.1% formic acid and 5 mM ammonium formate. MS analysis was performed with an electrospray source ionization (ESI) interface. The capillary voltage was set to 3.0 kV and the fragmentor voltage to 100 V. The drying gas temperature was 350°C, the drying gas flow was 10 L/min, and the nebulizer pressure was 45 psi. Data was collected using a mass range from 100–1200 Da.

### Statistical analysis

For luciferase reporter experiments all results were performed in triplicate and are reported as mean +/− SD. For RT-PCR, results are reported as mean +/− SD. Differences were analyzed by 2 tailed, student's t test. LC/MS data are presented as mean +/− SD of the ion intensity area. Results are interpreted as statistically significant for p value<.05.

## Results

### Hepatic Lipase uses VLDL preferentially to activate PPARδ

**Figure 1:**
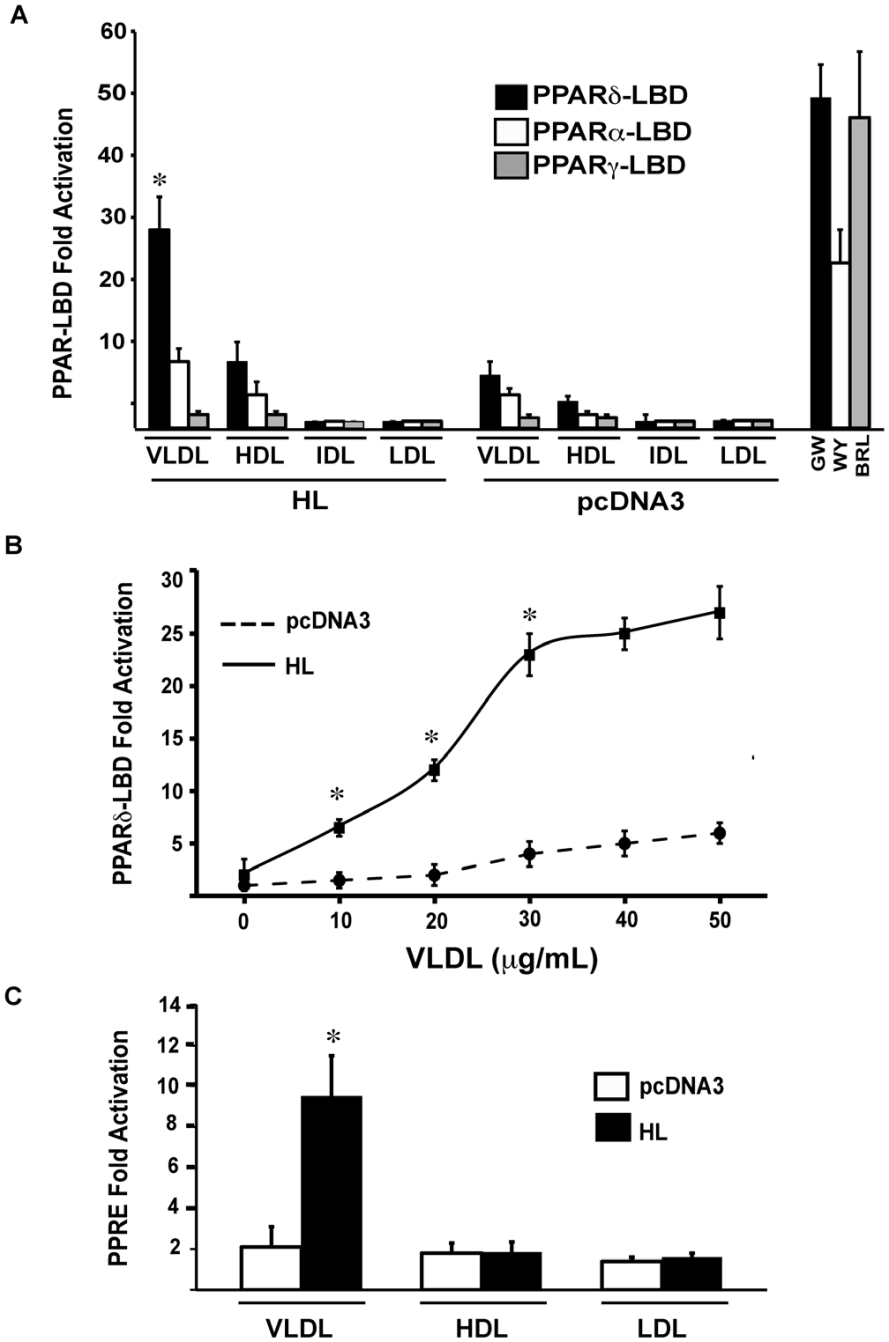
Hepatic lipase activates PPARδ selectively in the presence of human VLDL. (A) HEK293 cells transfected with human PPARα, γ, or δ ligand binding domain-Gal4 fusion constructs along with the pUAS×4TK Gal4 dependent luciferase reporter were stimulated with human VLDL 50 µg/mL, IDL 20 µg/mL, HDL 100 µg/mL or LDL 100 µg/mL for 12 hours. Luciferase values were normalized to β-galactosidase and results expressed as mean fold change of the normalized luciferase RLU. Each experimental condition was performed in triplicate and results are mean +/− SD. *p<.05 vs. empty vector. (B) HEK-293 cells were transfected with the PPARδ-LBD along with the luciferase reporter above. Cells were stimulated with indicated concentrations of human, pooled VLDL for 12 hours, then samples harvested for analysis. * p<.05 vs. empty vector. (C) HEK-293 cells were transfected with a PPRE-Luciferase reporter along with CMV-β-galactosidase and pcDNA or HL. Following transfection cells were stimulated with VLDL 50 µg/mL, HDL 100 µg/mL or LDL 100 µg/mL as before for 12 hours. *p<.05 vs. pcDNA.

Standard PPAR ligand binding domain (LBD) assays for all three human PPAR isoforms were performed in hepatic lipase (HL)-transfected 293 cells stimulated with HDL, IDL, LDL and VLDL at the concentrations shown (normalized to total protein). With TG-rich VLDL as a substrate, HL activated PPARδ preferentially relative to PPARα or PPARγ, with significantly lesser responses to HDL and even less for LDL and IDL (VLDL>>>HDL>LDL, IDL, Fig. 1A). HDL stimulation with protein concentrations as high as 1 mg/mL, which approximates circulating Apolipoprotein A1 plasma levels, had no effect on PPAR activation (Fig. S3). PPARδ LBD activation in HL-expressing cells was evident in a VLDL concentration-dependent manner (10–50 µg/mL, Fig. 1B). In similar HL-transfected 293 cells, VLDL stimulation also activated a co-transfected canonical PPAR response element (PPRE) direct repeat luciferase construct (Fig. 1C). HDL, IDL or LDL stimulation had no effect on the PPRE-reporter in similar HL-transfected cells (Fig. 1C).

### HL/VLDL activation of PPARδ requires intact catalytic HL function

**Figure 2:**
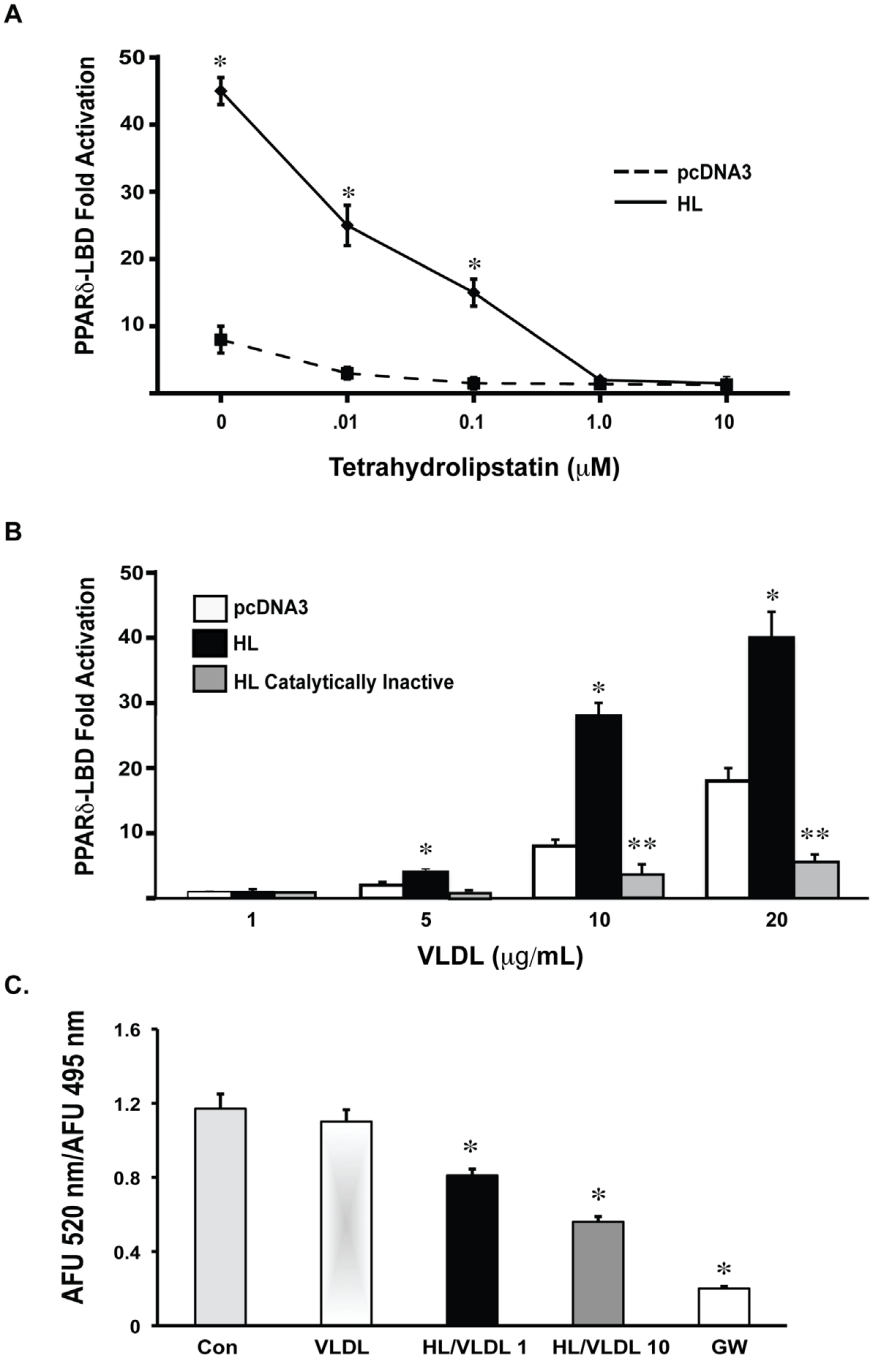
HL/VLDL activation of PPARδ depends on catalytic function. (A) HEK-293 cells were transfected with PPARδ-LBD and pretreated with indicated concentrations of tetrahydrolipstatin (THL) or DMSO control for 30 minutes prior to stimulation with VLDL 30 µg/mL for 12 hours. Results expressed as mean fold change. *p<.05 vs. empty vector. (B) COS-7 cells were transfected with pcDNA, HL or catalytically inactive HL and stimulated with indicated concentrations of VLDL for 12 hours. *p<.05 vs. pcDNA. **p<.05 versus HL and pcDNA. (C) Time-resolved FRET PPARδ competitive binding assay: Recombinant HL was incubated with increasing concentrations of VLDL (1 µg/mL and 10 µg/mL) for 1 hour with recombinant GST-PPARδ-LBD, terbium-labeled anti-GST antibody and a fluorescein-labeled PPARδ synthetic agonist. FRET activity, measured by the emission ratio of fluorescence at 520 nm/495 nm, was inhibited in a VLDL concentration dependent manner in the presence of HL. The synthetic PPARδ ligand, GW501516, maximally inhibited FRET at 1 µM. *p<.05 compared to VLDL alone.

In addition to its lipolytic activity, HL can also promote non-catalytic lipoprotein transport into cells [33], [34]. The role of HL catalytic function in PPAR activation was investigated in two ways: using the lipase inhibitor tetrahydrolipstatin and transfection of a catalytically inactive HL mutant. Tetrahydrolipstatin inhibited HL/VLDL activation of PPARδ in a dose-dependent manner in 293 cells (Fig. 2A). In a complementary approach, we mutated the catalytic site in human HL by site-directed mutagenesis. This mutant expressed to a similar degree in cell lysate and media compared to wild type HL, as determined by western blot (Fig. S1A), but possessed no enzymatic activity (Fig. S1B). In the presence of VLDL stimulation, catalytically inactive HL no longer activated PPARδ (Fig. 2B). Together, these data establish HL-mediated PPARδ activation requires HL catalytic function. To further examine the direct role of HL hydrolysis of VLDL in generating a PPARδ ligand, we performed time-resolved, fluorescence resonance energy transfer (TR-FRET) PPARδ competitive binding assays with increasing concentrations of VLDL in the presence of recombinant HL. This cell free assay measures FRET induced by interaction between a known, fluorescently labeled synthetic PPARδ agonist and recombinant, fluorescently labeled PPARδ LBD. The presence of another PPARδ ligand, in this case one generated by HL-mediated VLDL hydrolysis, causes competitive displacement of the labeled ligand resulting in decreased FRET. When tested in this cell free system, recombinant HL in the presence of VLDL reduced FRET in a linear, VLDL concentration-dependent manner, consistent with a concentration-dependent increase in a PPARδ ligand (Fig. 2C). These results establish that HL hydrolysis of VLDL is sufficient to generate a PPARδ agonist.

### HL/VLDL induces expression of ADRP, a canonical PPARδ target gene, in a manner dependent on HL catalysis and PPARδ expression

**Figure 3:**
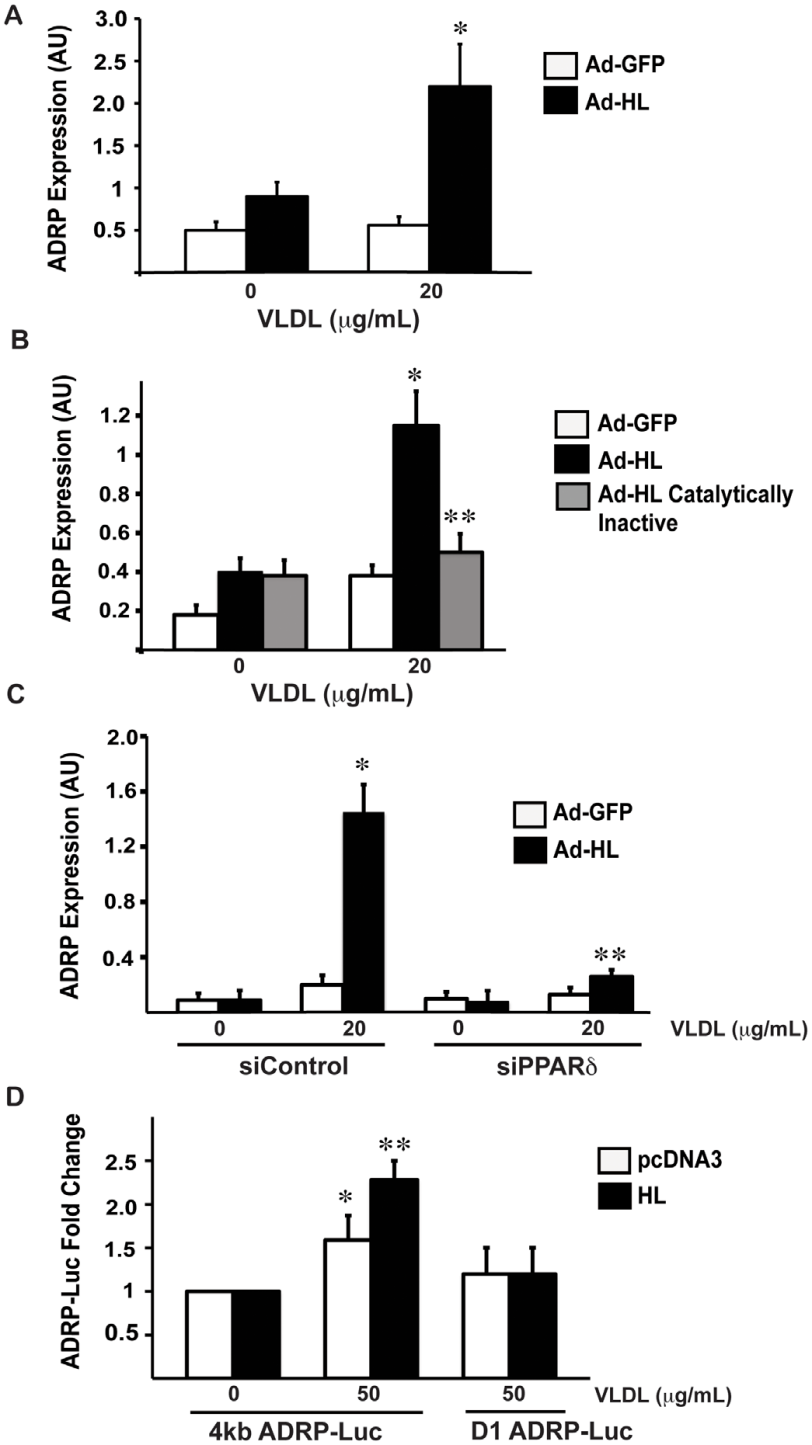
HL/VLDL increases expression of the canonical PPARδ target gene, ADRP. (A) HUVEC were infected with adenovirus constructs expressing GFP or human HL (MOI of 100) in growth medium for 24 hours. The medium was replaced with serum free medium with lipoprotein stimulation where indicated for an additional 12 hours. RT-PCR results are expressed as relative copy number arbitrary units (AU), as the ratio of ADRP to 36B4 internal control. *p<.05 vs. GFP. (B) HUVEC were infected as above with adenovirus constructs expressing GFP, HL or catalytically inactive HL for 24 hours. Cells were then stimulated with VLDL 20 µg/mL for 12 hours in serum free medium and RNA harvested for gene expression analysis. *p<.05 vs. GFP. ** p<.05 vs. HL. (C) HUVEC were transfected with the indicated siRNA constructs for 24 hours. Following transfection, cells were infected with adenovirus constructs for 24 hours in HUVEC growth medium. After adenovirus infection, cells were stimulated with VLDL for an additional 12 hours before harvesting RNA for gene expression analysis. *p<.05 vs. GFP, **p<.05 vs HL. (D) COS-7 cells were transfected with either the 4 kb-ADRP luciferase reporter, containing the endogenous PPRE from the human ADRP promoter, or the D1-ADRP luciferase reporter, possessing a point mutation in the PPRE. Where indicated cells were also transfected with pcDNA3 or HL and stimulated with VLDL 50 µg/mL for 12 hours. Luciferase and β-galactosidase were harvested as before. *p<.05 vs. control **p<.05 vs. control/VLDL.

PPARδ regulates genes involved in lipid metabolism. Adipocyte differentiation-related protein (ADRP), a lipid droplet associated protein involved in the cellular adaptation to lipid loading, is an established PPARδ target gene [31]. We used primary human umbilical vein endothelial cells (HUVEC) as a heterologous cell model that contains PPARδ but does not express HL or LPL to test whether VLDL regulates ADRP gene expression after adenoviral HL (Ad-HL) versus control GFP (Ad-GFP) infection. VLDL stimulation (20 µg/mL) of Ad-HL infected HUVECs increased ADRP mRNA levels ∼4 fold but had no effect on Ad-GFP cells, as assessed by RT-PCR (Fig. 3A). A similar fold change in ADRP mRNA expression was observed with the PPARδ synthetic agonist GW501516 (1 µM) in HUVECs. In contrast, adenoviral transfection of a catalytically inactive HL had no effect on ADRP expression in the presence of VLDL stimulation in this same HUVEC system (Fig. 3B). To test if HL/VLDL induction of ADRP was dependent on PPARδ, similar experiments were repeated in the presence of a small interfering PPARδ construct (siPPARδ, Fig. S2). ADRP mRNA expression in response to HL/VLDL stimulation was decreased ∼80% in the presence of siPPARδ versus control siRNA (Fig. 3C). Previous work has identified a functional PPRE in the human ADRP promoter region (−2361 to −2345 bp) [31]. To determine if HL/VLDL activates ADRP expression through this PPRE, we used a 4 kb human ADRP promoter-luciferase reporter construct (4 kb ADRP-Luc) transfected into COS cells. VLDL stimulation of COS cells expressing HL doubled the activity of the cotransfected ADRP promoter-Luc reporter; no effect was seen in an ADRP promoter construct in which the PPRE contained a point mutation (D1 ADRP-Luc, Fig. 3D).

### HL/VLDL regulates PPARδ target gene expression in FAO hepatoma cells *in vitro* and in murine liver *in vivo*

**Figure 4:**
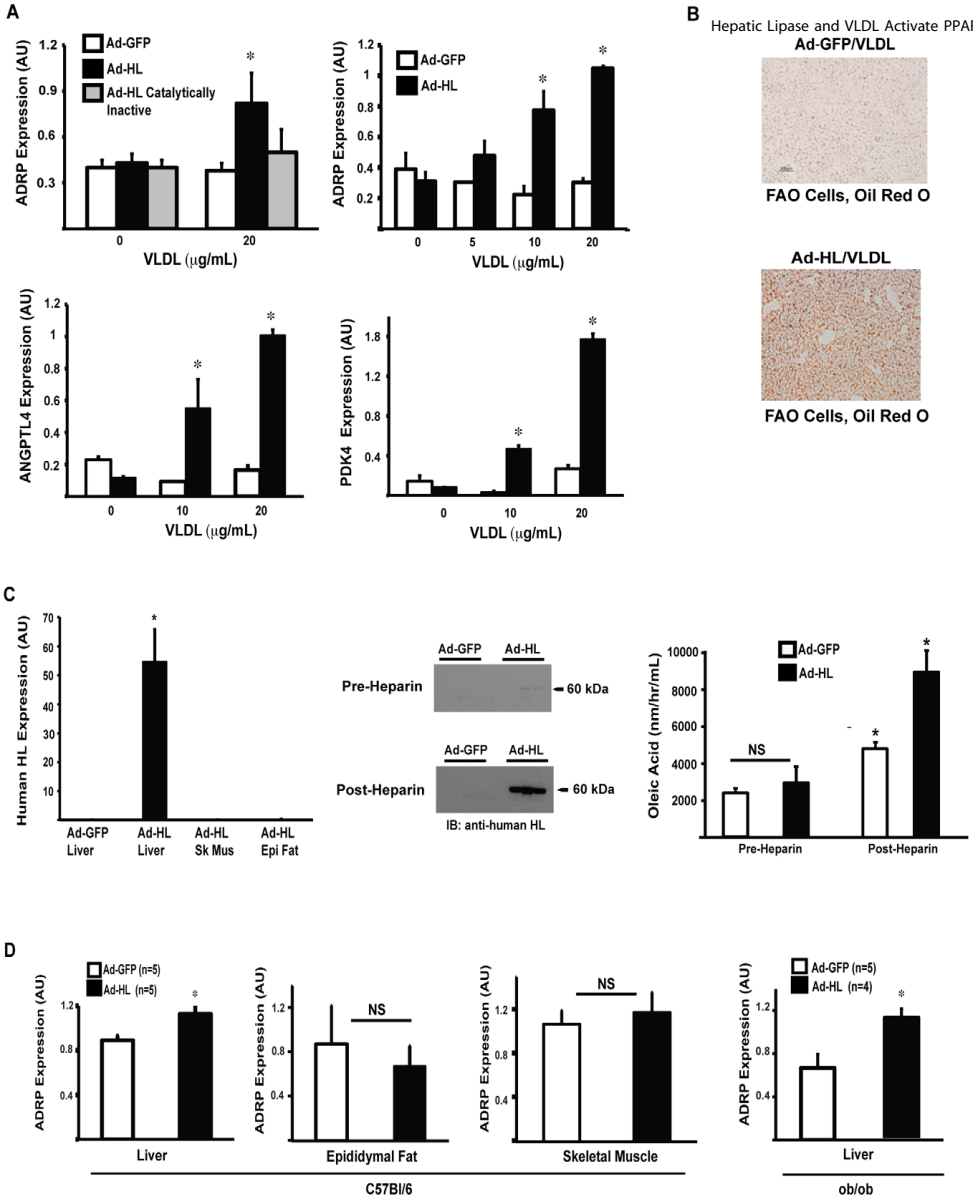
HL/VLDL increases expression of PPARδ target genes, *in vitro* and *in vivo*. (A) Left Panel. FAO hepatoma cells were infected with adenovirus GFP, adenovirus HL and adenovirus catalytically inactive HL for 24 hours in serum free medium. After 12 hours stimulation with lipoprotein, RNA was harvested for gene expression analysis and results are expressed as before. *p<.05 vs. GFP. (A) Right Panel. The concentration dependent effect of VLDL induction of ADRP mRNA in HL-expressing FAO cells is shown. (B) Oil Red O staining. FAO hepatoma cells treated with adenovirus GFP and HL along with VLDL 50 µg/mL were fixed in paraformaldehyde overnight then stained with Oil Red O to mark neutral lipid accumulation. Magnification 10×. (C) C57BL/6 (8 week, males) were injected by tail vein with adenovirus GPF or human HL constructs, 1×10^11^ viral particles, 100 µL volume. Five days following adenovirus infection, plasma was collected pre-heparin and 30 minutes post heparin injection (intraperitoneal, 200 U) for HL activity and protein expression; mice were euthanized and tissue was harvested for RNA isolation for gene expression analysis. *p<.05 vs. GFP. RT-PCR results expressed as relative copy number in arbitrary units. (D) C57BL/6 and ob/ob mice (6–8 week, male) underwent tail vein injection as above. Five days later tissue was harvested for gene expression. *p<.05 vs. GFP. RT-PCR results expressed as relative copy number in arbitrary units.

Given the data above, we next tested HL regulation of ADRP expression through PPARδ in a hepatocyte-derived model system *in vitro*. Rat FAO hepatoma cells were used since primary mouse hepatocytes lose PPARδ expression shortly after isolation (data not shown). VLDL stimulation of FAO hepatoma cells infected with Ad-HL significantly increased ADRP mRNA levels in a VLDL concentration-dependent manner (Fig. 4A). HL hydrolysis of VLDL also increased expression of angiopoietin like protein-4 (ANGPLT4) and pyruvate dehydrogenase kinase 4 (PDK4), two known PPARδ-regulated genes with important roles in systemic lipid metabolism (Fig. 4A) [31]. ADRP facilitates storage of neutral lipids in cytoplasmic lipid droplets. Consistent with this role, HL/VLDL stimulation increased lipid accumulation in FAO cells versus control, as measured by Oil Red O staining (Fig. 4B).

We next investigated evidence for this HL/VLDL/PPARδ pathway *in vivo*. Although human and murine HL metabolize similar lipoprotein substrates, murine HL circulates freely in plasma in mice (>70% of HL activity), whereas human HL is retained in the liver, bound to heparin sulfate proteoglycans (HSPGs) on the hepatocyte cell surface [35]. Eight week old C57BL/6 male mice on standard chow diet were infected with similar human Ad-GFP or Ad-human HL constructs by tail vein injection, followed five days later by plasma and tissue harvest for analysis of human HL protein and activity as well as changes in gene expression. Human HL mRNA expression was restricted to the liver and was not detected in skeletal muscle or epididymal fat (Fig. 4C). HL protein levels and activity in the plasma were also not different compared to GFP expressing mice at baseline (Fig. 4C). However, thirty minutes after heparin treatment plasma HL activity was significantly higher in the human HL-overexpressing mice compared with the Ad-GFP injected mice. This result is consistent with human HL localization to liver HSPGs and release into plasma by heparin (Fig. 4C). As such, HL expression by adenoviral infection provides a model to test directly whether increasing hepatocyte HL expression regulates hepatic PPARδ transcriptional responses *in vivo*. In this model, ADRP expression was significantly increased by 30% in livers from the C57Bl/6 mice receiving HL as compared to GFP (Fig. 4D). ADRP expression did not differ between HL or GFP in the epididymal fat or skeletal muscle of these mice (Fig. 4D). As noted, HL is an important determinant of circulating TG levels. In C57BL/6 mice, TG levels are low on standard chow diet. Adenoviral HL infection in C57BL/6 mice decreased TG levels from 48 mg/dL to 28 mg/dL. Given our *in vitro* data for VLDL, a TG-rich lipoprotein, serving as the preferred substrate for HL-mediated PPARδ activation, we reasoned that circulating TG levels might influence HL/VLDL mediated PPARδ responses. The *ob/ob* mouse develops modestly higher plasma TGs than chow-fed C57BL/6 mice, but still within the normal range. Six-eight week old, *ob/ob* mice were infected with Ad-GFP or Ad-HL. Baseline TG levels were 70 mg/dL the Ad-GFP group versus 25 mg/dL in the Ad-HL cohort five days after adenoviral infection. In this ob/ob model with higher triglycerides compared to the wild-type mice, hepatic ADRP mRNA levels increased more than in the C57BL/6 mice (70% vs 30%) following HL overexpression, as compared to GFP (Fig. 4D, Liver – ob/ob).

### VLDL hydrolysis by HL liberates unsaturated FAs that activate PPAR transcription

HL/VLDL activates PPARδ through its catalytic products. VLDL is a TG-rich lipoprotein, with lower concentrations of PL, diacylglycerol, monoacylglycerol and cholesterol [36]. To investigate the role of specific HL/VLDL hydrolytic products in PPARδ activation, a global metabolite profiling approach using liquid chromatography coupled to mass spectrometry (LC/MS) was employed [37]. A profiling LC/MS approach can identify metabolites generated by HL-mediated VLDL hydrolysis in an unbiased fashion. By coupling this global metabolite profiling approach with our prior experimental data and methods, the ability of specific HL/VLDL metabolites to activate PPARδ can be tested. Further, by using ^13^C labeled oleic acid as an internal standard, LC/MS also enables quantification of specific FA products liberated by HL/VLDL.

**Figure 5:**
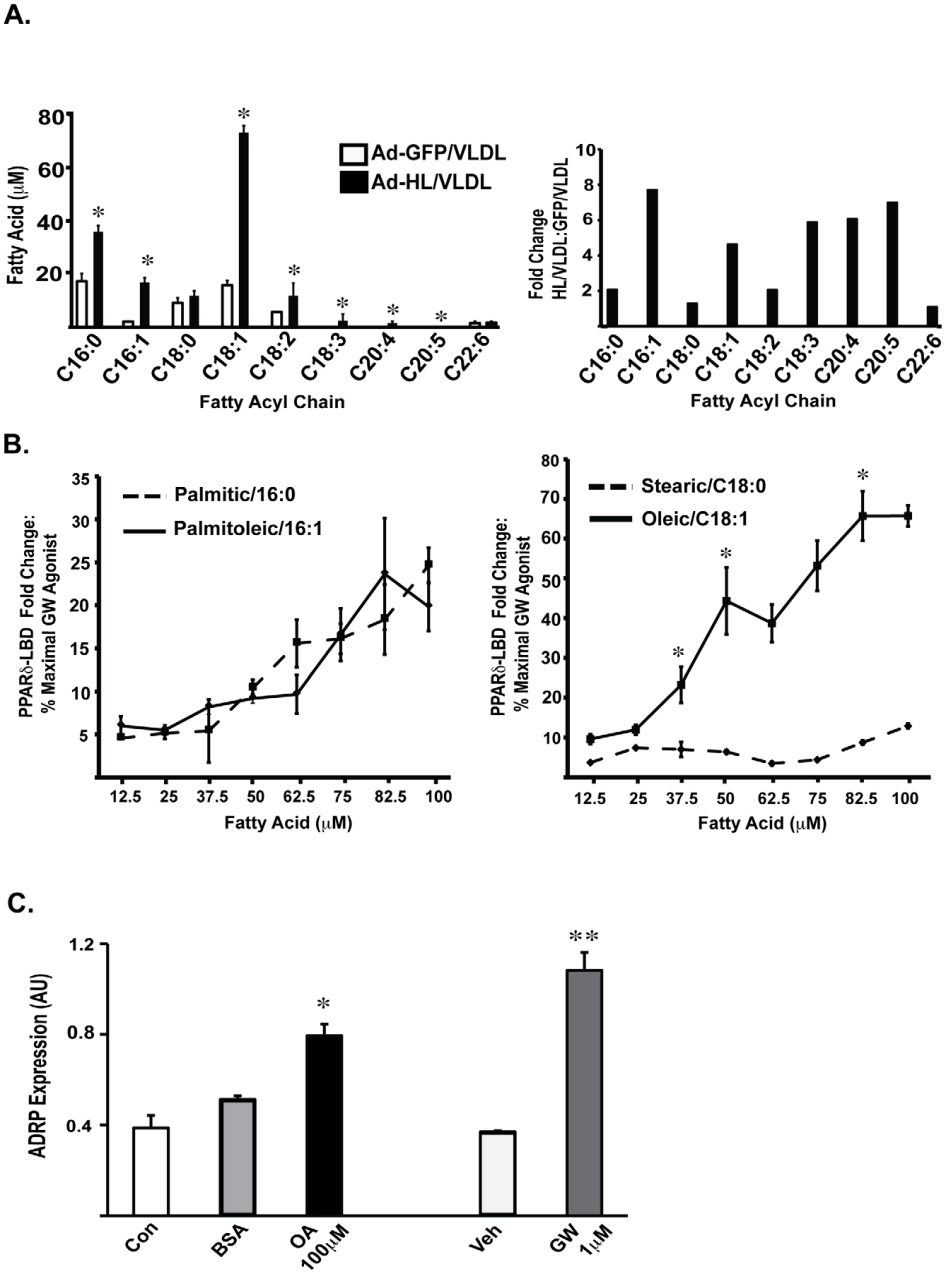
Global metabolite profiling identifies specific unsaturated FAs generated by HL hydrolysis of VLDL that activate PPAR transcriptional responses. (A) Left Panel. Negative ionization mode LC/MS analysis of lipid extracts from HL/VLDL treatment group demonstrated significant increases in levels of long chain fatty acids including palmitic (C16:0), palmitoleic (C16:1), oleic (C18:1), linoleic (C18:2), linolenic (C18:3), arachidonic (C20:4), eicosapentaenoic (C20:5) and docosohexaenoic (C22:6) acid. Absolute nanomoles of fatty acid were calculated using the integrated ion intensity of the internal standard C^13^ oleic acid. Each experimental condition was performed in triplicate, with results representing the mean +/− SD. Right Panel. The absolute fold change for each fatty acid comparing treatment with HL/VLDL vs GFP/VLDL is shown. *p<.05 vs GFP/VLDL for each fatty acid. (B) COS-7 cells were transfected with PPARδ-LBD and stimulated with the fatty acids fatty acids indicated. Results expressed as the percent of maximal LBD activation measured with PPARδ synthetic agonist GW501516. *p<.05 vs control. (C) FAO cells were treated with indicated concentrations of fatty acid in media with 1% BSA, BSA control or the PPARδ synthetic agonist GW501516 at maximal concentration. Following stimulation RNA was harvested and ADRP expression analyzed by RT-PCR. Results expressed as relative copy number in arbitrary units. *p<.05 vs BSA and control. **p<.05 vs vehicle.

As compared to GFP-expressing COS cells, VLDL stimulation of HL-expressing cells (6 hours) generated statistically significant increases in levels of specific long chain FA species, as measured by LC/MS in negative ionization mode (Fig. 5A, left panel). This platform also affords analysis of relative TG versus PL hydrolysis by HL with intact lipoprotein substrates. Sample analysis in positive ionization mode revealed greater reductions of both TG and diacylglycerol levels relative to PL levels in the HL group, indicating TG as the major source of HL-generated FA in this model (Table 1). Other lipids, including cholesterol, cholesterol esters and sphingomyelin, were unchanged (Table 1).

**Table 1:** Relative Levels of Representative Lipids Measured by Metabolite Profiling in Samples with HL∶VLDL and GFP∶VLDL^a^.

Lipid Class	HL∶VLDL/GFP∶VLDL
Phospholipids	
C34:1 PC	0.83
C34:2 PC	0.73*
C36:2 PC	0.70*
C36:3 PC	0.66*
TAGs	
C48:2	0.34*
C50:2	0.40*
C54:6	0.34*
DAGs	
34:1	0.10*
36:2	0.09*
LPCs	
C18:1	4.2*
CEs	
C18:1	0.97
C18:2	0.52
SM	
C16:0	0.81
C16:1	0.89
FFAs	
C16:0	(2.1)*
C16:1	(7.7)*
C18:1	(4.6)*
C20:4	(6.1)*
C20:5	(7.0)*
C22:6	(1.1)

a Data in parentheses were determined by directly quantifying FA levels using targeted LC/MS method with internal FA standard. TAGs, triacylglycerols; DAGs, diacylglycerols; LPCs, lysophosphatidic acid; CEs, cholesterol esters; SM, sphingomyelin; FFAs, free fatty acids. Data represent the mass ion intensity ratios performed in triplicate for each experimental condition.

*p<.05 for HL∶VLDL compared to GFP∶VLDL.

The fold changes seen in HL- versus GFP-expressing cells for both saturated and unsaturated FAs were significant, but a proportionally larger difference in release of monounsaturated and polyunsaturated FAs was detected, with a particular difference in palmitoleic (PO - C16:1, 8 fold) and oleic (OA - C18:1, 4.5 fold) FAs (Fig. 5A, right panel). The fold changes for linoleic (C18:2), linolenic (C18:3), arachidonic (C20:4), and eicosapentaenoic (C20:5) were also statistically significant (Fig. 5A, right panel).

We focused on the most abundant FAs identified by metabolite profiling, PO and OA (15, 80 µM, Fig. 5A, left panel), as well as the corresponding saturated FAs palmitate (PA, C16:0) and stearate (SA, C18:0) to determine if the specific HL/VLDL-induced FA changes detected were relevant for PPARδ activation. Both PA and PO modestly activated PPARδ LBD, achieving 25% LBD activation at 100 µM relative to maximal stimulation with the potent PPARδ selective agonist GW50156. By contrast, OA activated PPARδ in a concentration-dependent fashion, achieving approximately 70% of maximal GW activation at 100 µM (Fig. 5B); SA only modestly activated PPARδ at the same concentration. Additionally, the polyunsaturated FAs (C18:2, C18:3, C20:4, C20:5 and C22:6) demonstrated minimal PPAR transcriptional activity when used at the concentrations quantified in metabolite profiling experiments (data not shown). Given evidence that OA but not SA was released by HL/VLDL hydrolysis, we next returned to ADRP regulation. We tested if OA regulates ADRP gene expression in FAO hepatoma cells. OA stimulation (12 hrs, 100 µM) increased ADRP mRNA expression two fold, ∼70% of the GW501516 response and consistent with the PPARδ LBD assay responses (Fig. 5C).

## Discussion

The nuclear receptors PPARα, δ and γ coordinate carbohydrate, lipid, and lipoprotein metabolism. Despite the importance of these transcription factors in hepatocyte biology, mechanisms of PPAR activation in the liver remain poorly understood. The data provided here identifies a new role for hepatic lipase (HL) in PPAR activation. HL hydrolyzes VLDL to generate predominantly PPARδ activation, with markedly lesser effects on PPARα and even less on PPARγ. Despite HL's known phospholipase activity, HDL, the most PL-rich circulating lipoprotein class, was less effective as a substrate in HL-mediated PPARδ activation as compared to VLDL. IDL remnant lipoprotein exposure to HL also did not activate PPARδ. In support of this novel HL/VLDL/PPARδ pathway, HL/VLDL induced expression of the canonical PPARδ target genes ADRP, ANGPLT4 and PDK4, in HUVECs and FAO cells, and had functional effects on increased lipid droplet formation. In keeping with these findings, hepatic expression of human HL *in vivo* increased ADRP expression in C57BL/6 and ob/ob mice. Global metabolite profiling to annotate lipid products of HL hydrolysis of VLDL revealed proportionally more release of monounsaturated (MUFA) and polyunsaturated (PUFA) fatty acids including OA and PA. Consistent with this finding, direct OA stimulation of FAO cells *in vitro* recapitulated PPARδ activation by HL/VLDL, inducing ADRP expression, while PA had only modest PPARδ effects; stearic acid, which was not released by HL action on VLDL, had no PPARδ effects.

LPL, EL and HL are members of a family of lipases that hydrolyze lipid substrates like TGs and PLs. Multiple parameters define the distinct function of these lipases, including their expression patterns, substrate preferences, catalytic activity, endogenous inhibitors and cofactors [38]. Despite their critical role in lipoprotein metabolism, specific links between the action of these lipases and fatty acid-activated nuclear receptor responses remain poorly defined. Prior work suggests lipases can induce PPAR responses, although mainly with selective PPARα activation. Our group reported that LPL and EL could activate PPARα through mechanisms involving VLDL and HDL, respectively [11], [12]. Others have shown that LPL-overexpression in cardiac myocytes also induces PPARα target gene expression [14]. Chawla et al. reported LPL-mediated PPARδ regulation in murine macrophages, which express lower levels of PPARα [28]. Both murine and human hepatocytes express PPARα and PPARδ, making HL's preferential activation of PPARδ over PPARα by the same VLDL substrate noteworthy. VLDL is a TG rich lipoprotein; PPAR activation using VLDL as a substrate implicates TG as an important source for PPAR ligands formed by HL-mediated catalysis. The relative inability of HDL, IDL and LDL to activate PPAR responses in our experimental models may be a consequence of the lower TG content of that characterizes these other lipoprotein classes *in vivo*. Additional studies will be needed to determine how other factors such as apolipoprotein composition, circulating cofactors or other cell surface co-receptors may also influence lipase-lipoprotein mediated PPAR activation.

LPL, EL and HL can also facilitate lipoprotein transport independent of their enzymatic activity [34], [38]. We demonstrate that HL-mediated PPARδ activation requires active catalysis. Although a catalytically inactive HL mutant can promote cellular lipoprotein uptake, it was no longer capable of activating PPARδ. Similarly, a general lipase inhibitor also repressed HL-mediated PPARδ activation. In a TR-FRET ligand displacement assay, VLDL hydrolysis by HL displaced a fluorescently labeled ligand within one hour in a VLDL concentration-dependent way. Although other intracellular enzymes could conceivably metabolize VLDL following its endocytosis resulting in PPARδ activation, the data presented here argues for hydrolysis of VLDL and release of metabolites by HL positioned on the cell surface as a major contributor to PPAR activation. Together these results suggest specific products of HL catalysis of VLDL may account for PPARδ activation.

Metabolite profiling using liquid chromatography coupled with mass spectrometry (LC/MS) is a robust method for annotating lipid metabolites produced by enzymes in complex biological systems [37], [39]. By applying this unbiased strategy to analyze lipids generated by HL-mediated hydrolysis of VLDL, specific molecules released by HL/VLDL were identified, including ones that could replicate HL/VLDL/PPARδ effects. Several new insights have emerged using this approach. First, VLDL hydrolysis by HL liberated OA and PA at micromolar concentrations, levels that overlap the concentrations needed for PPAR activation, as determined in concurrent PPAR transactivation assays. Second, other FAs including linolenic, arachidonic, eicosapentaenoic, and docosohexaenoic acid were also released by HL, but did not stimulate PPAR transactivation, at least within this system. Third, the HL depleted TG levels of VLDL more than PLs, further demonstrating HL's important TG hydrolase function and highlighting the role of TG as a substrate in HL-mediated PPARδ activation. It will be of interest to apply this system to investigate the lipid metabolites generated by LPL and EL, which are also relevant for isotype specific PPAR responses.

Our finding here that OA is a major FA product of HL lipolysis that can activate PPARδ aligns with several recent reports in the literature. Sanderson et al. found that OA activates PPARδ, not PPARα responses in liver. In those studies the source of OA production was not explored [40]. Our data identifies HL as a specific pathway relevant for hepatocytes that can generate OA from VLDL, thus offering a direct link between hepatic lipid metabolism and PPARδ activation via HL-generated OA. Of note, the hydrophobic nature of FAs liberated by HL requires cytosolic chaperones such as fatty acid binding proteins (FABPs) to facilitate FA delivery to the nucleus. In this regard, Tan et al. reported that FABP5 binds OA and translocates this FA to the nucleus where it interacts with PPARδ, thereby promoting keratinocyte differentiation [41]. By integrating unbiased lipoprotein/HL/PPAR activation assays, cell biology studies, and LC/MS analysis of HL lipolytic reactions, the data provided here converge with these observations using completely different approaches. Collectively, these findings suggest a model involving integrated extracellular to intracellular to nuclear metabolic pathways that determine specific transcriptional responses. Similar, but distinct pathways may exist for other nuclear receptors as defined by variables of lipoprotein substrates, lipases, metabolites, surface receptors and/or FABPs.

Lipolytic mechanisms that direct specific PPAR isotype activation suggests new ways to consider how lipid metabolism may couple to transcriptional and cellular responses. Genetic and synthetic agonist studies establish a key role for PPAR in the transcriptional regulation of genes involved in lipogenesis and lipid storage typical of the fed state [42]. In contrast, PPARα controls a transcriptional program regulating fatty acid β-oxidation and ketogenesis in the liver as encountered during fasting [43]. The action of HL to induce PPARδ-mediated lipogenesis, including lipid droplet formation through ADRP, mirrors PPARδ effects seen with synthetic agonists. In the liver, PPAR isotype-specific responses may also be controlled by metabolites formed by an opposing pathway of lipid metabolism. In support of this, Chakravarthy et al. recently identified that fatty acid synthase (FAS), the rate-limiting enzyme in hepatic *de novo* lipogenesis, could generate a specific phosphatidylcholine (GPC 16:0/18:1) capable of acting as an endogenous hepatic PPARα ligand [10]. The fact that FAS is the first committed step in palmitic acid biosynthesis but ultimately fosters FA oxidation through PPARα, while HL, as a triacylglycerol hydrolase/ phospholipase, functions in lipoprotein catabolism, but promotes lipogenesis through PPARδ suggests cross-integration of these pathways. Interestingly, levels of this GPC 16:0/18:1 ligand were modestly reduced in our experiments, entirely consistent with HL's phospholipase activity and revealing other ways through which lipolysis may influence both PPARδ and PPARα responses. Based on the data presented here, it will be of interest to consider how HL-mediated PPARδ activation influences systemic and hepatic metabolic responses in vivo in the context of HL and PPARδ loss of function in mice and humans. The recent observation that HL-deficient mice are protected from diet-induced obesity, display altered energy substrate utilization and have decreased hepatic steatosis suggests that HL may play a broader physiologic role in metabolic adaptations to diet [44]. Further work will be needed to resolve these issues. The finding that VLDL hydrolysis by HL preferentially activates PPARδ provides a new way to reconsider the functional role of HL and PPARδ in the liver and serves as an example of how cell-based assays can be combined with global metabolite profiling to explore how lipid metabolism modulates transcriptional responses.

## Supporting Information

Figure S1 **Catalytic mutant HL expresses similar protein levels, but has no triglyceride hydrolase activity.** Left Panel. Control vector, HL or HL catalytic mutant expression vectors were transfected into COS cells. 24 hours after transfection, heparin (10 Units) was added to the media to release the lipase from the cell surface. Both media and cell lysate were collected for western blot. Right Panel. Media collected from above was used in triolein activity assay.
(TIF)

Figure S2 **SiRNA knockdown of PPARδ achieved an 80–90% knockdown of PPARδ expression.** HUVEC were transfected with siRNA for PPARδ or a scrambled control. Following lipoprotein stimulation, RNA was collected for analysis of gene expression. *p<.05 for siPPARδ versus siControl.
(TIF)

Figure S3 **Physiologic HDL concentrations activate PPARδ, but not to the same degree as VLDL.** COS cells were transfected with each of the human PPAR-LBDs and stimulated with HDL (1 mg/mL) as in Figure 1. Data are presented as relative fold change of luciferase/β-galactosidase.
(TIF)
