# Supplementary figures and images for: VLDL Hydrolysis by Hepatic Lipase Regulates PPARδ Transcriptional Responses
Source: PLoS One. 2011 Jul 5;6(7):e21209. doi: 10.1371/journal.pone.0021209 (PMC3130023; doi:10.1371/journal.pone.0021209)

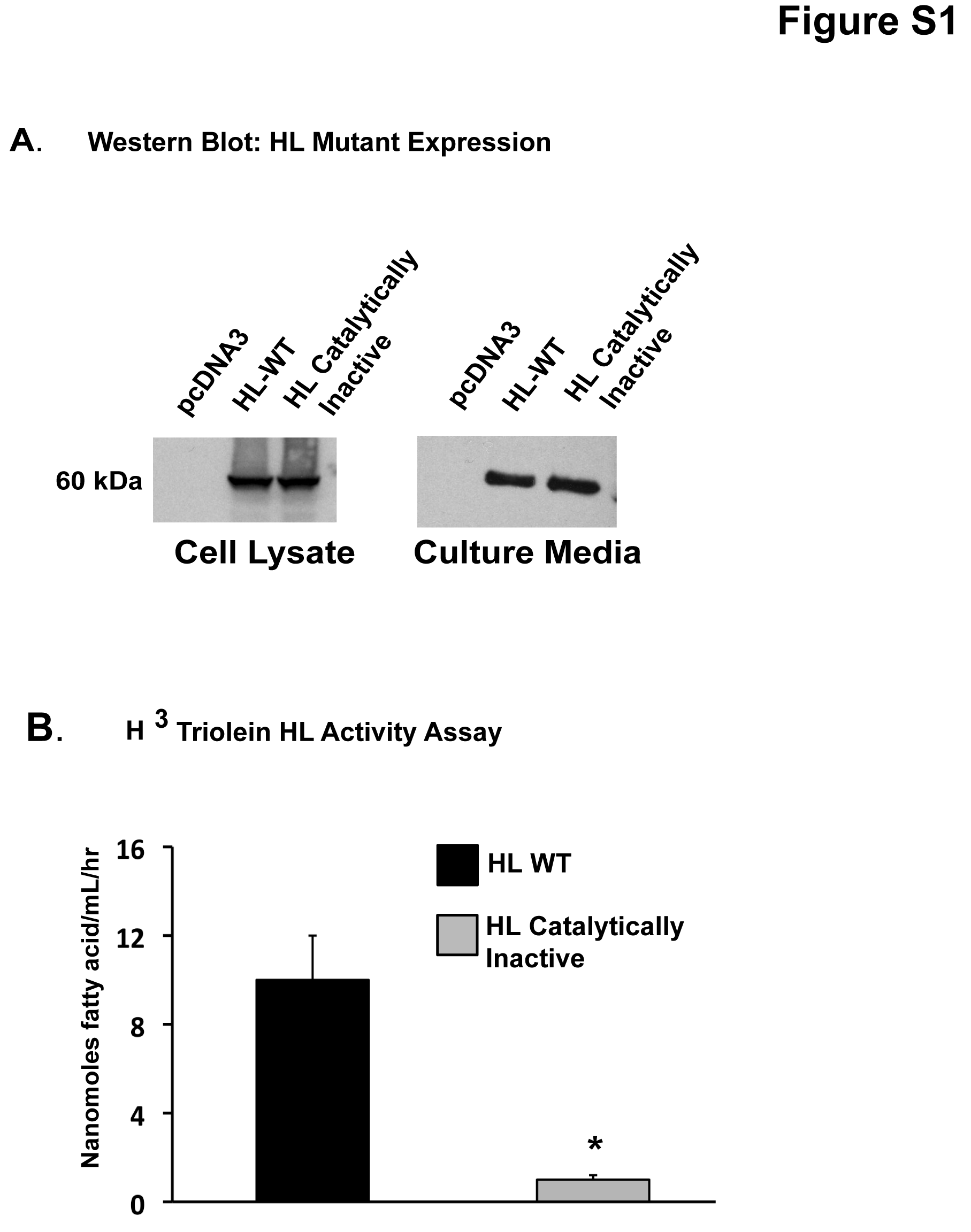

Supplement: Figure S1 — Catalytic mutant HL expresses similar protein levels, but has no triglyceride hydrolase activity. Left Panel. Control vector, HL or HL catalytic mutant expression vectors were transfected into COS cells. 24 hours after transfection, heparin (10 Units) was added to the media to release the lipase from the cell surface. Both media and cell lysate were collected for western blot. Right Panel. Media collected from above was used in triolein activity assay. (TIF) [file pone.0021209.s001.tif]

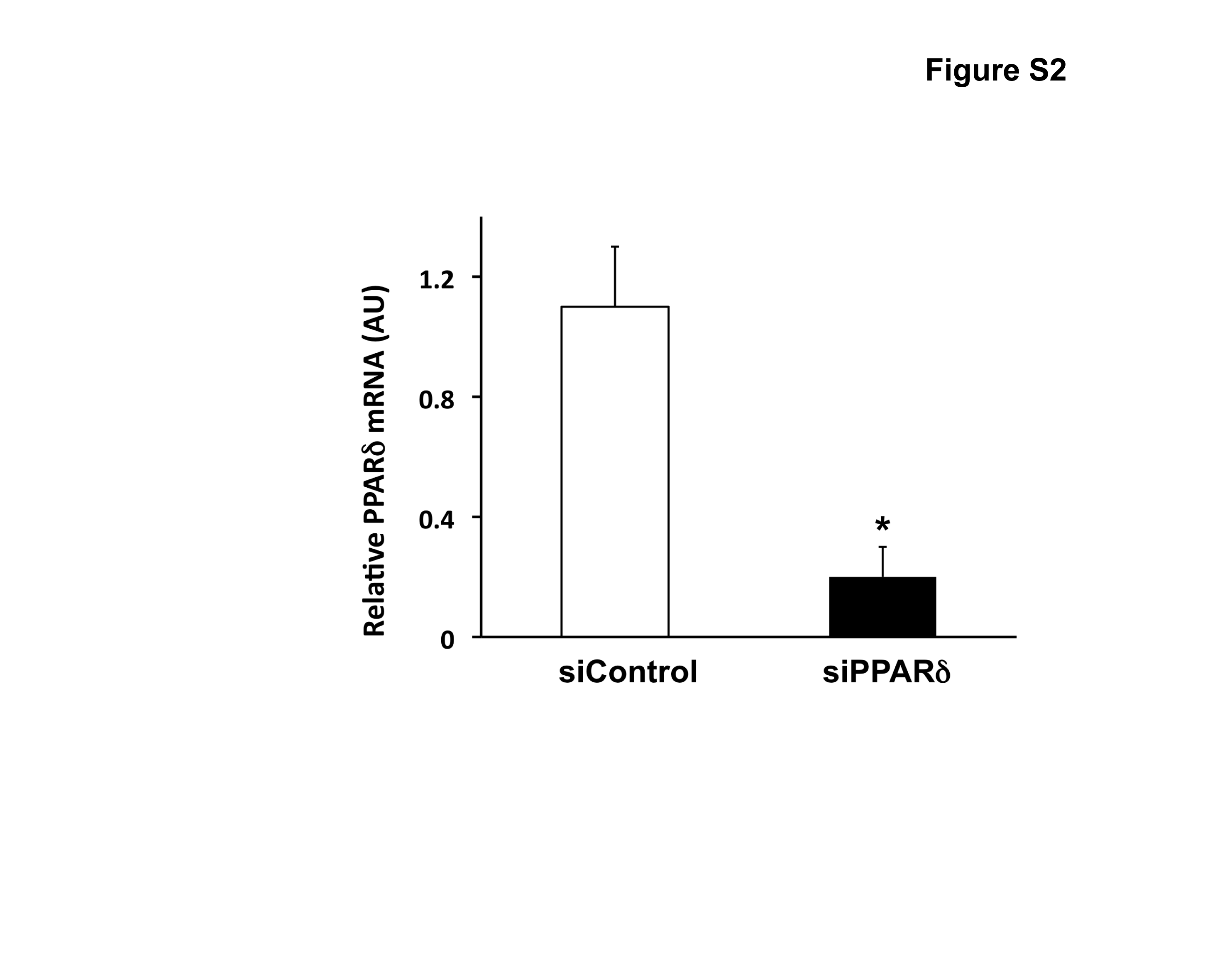

Supplement: Figure S2 — SiRNA knockdown of PPARδ achieved an 80–90% knockdown of PPARδ expression. HUVEC were transfected with siRNA for PPARδ or a scrambled control. Following lipoprotein stimulation, RNA was collected for analysis of gene expression. *p<.05 for siPPARδ versus siControl. (TIF) [file pone.0021209.s002.tif]

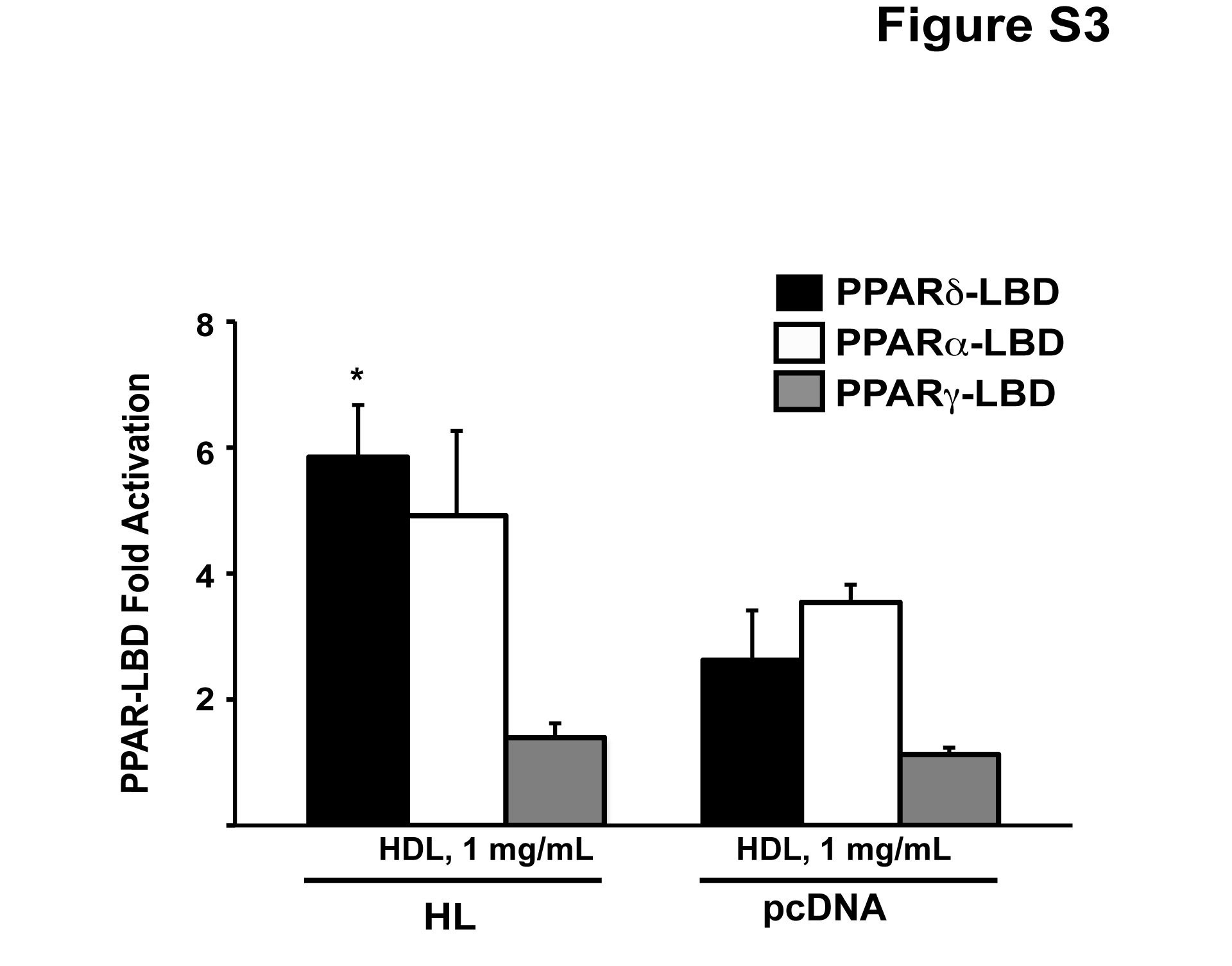

Supplement: Figure S3 — Physiologic HDL concentrations activate PPARδ, but not to the same degree as VLDL. COS cells were transfected with each of the human PPAR-LBDs and stimulated with HDL (1 mg/mL) as in Figure 1. Data are presented as relative fold change of luciferase/β-galactosidase. (TIF) [file pone.0021209.s003.tif]
